# Mitochondrial H_2_O_2_ as an enable signal for triggering autophosphorylation of insulin receptor in neurons

**DOI:** 10.1186/1750-2187-8-11

**Published:** 2013-10-05

**Authors:** Nadezhda A Persiyantseva, Tatiana P Storozhevykh, Yana E Senilova, Lubov R Gorbacheva, Vsevolod G Pinelis, Igor A Pomytkin

**Affiliations:** 1 Department Cellular and Molecular Technology, Scientific Centre for Children’s Health, RAMS, Lomonosovsky prospect 2/62, 119991 Moscow, Russia; 2 Faculty of Biology, Lomonosov Moscow State University, 119991 Moscow, Russia; 3 Biosignal Ltd., M. Gruzinskaya 29-153, 123557 Moscow, Russia

## Abstract

**Background:**

Insulin receptors are widely distributed in the brain, where they play roles in synaptic function, memory formation, and neuroprotection. Autophosphorylation of the receptor in response to insulin stimulation is a critical step in receptor activation. In neurons, insulin stimulation leads to a rise in mitochondrial H_2_O_2_ production, which plays a role in receptor autophosphorylation. However, the kinetic characteristics of the H_2_O_2_ signal and its functional relationships with the insulin receptor during the autophosphorylation process in neurons remain unexplored to date.

**Results:**

Experiments were carried out in culture of rat cerebellar granule neurons. Kinetic study showed that the insulin-induced H_2_O_2_ signal precedes receptor autophosphorylation and represents a single spike with a peak at 5–10 s and duration of less than 30 s. Mitochondrial complexes II and, to a lesser extent, I are involved in generation of the H_2_O_2_ signal. The mechanism by which insulin triggers the H_2_O_2_ signal involves modulation of succinate dehydrogenase activity. Insulin dose–response for receptor autophosphorylation is well described by hyperbolic function (Hill coefficient, n_H_, of 1.1±0.1; R^2^=0.99). N-acetylcysteine (NAC), a scavenger of H_2_O_2_, dose-dependently inhibited receptor autophosphorylation. The observed dose response is highly sigmoidal (Hill coefficient, n_H_, of 8.0±2.3; R^2^=0.97), signifying that insulin receptor autophosphorylation is highly ultrasensitive to the H_2_O_2_ signal. These results suggest that autophosphorylation occurred as a gradual response to increasing insulin concentrations, only if the H_2_O_2_ signal exceeded a certain threshold. Both insulin-stimulated receptor autophosphorylation and H_2_O_2_ generation were inhibited by pertussis toxin, suggesting that a pertussis toxin-sensitive G protein may link the insulin receptor to the H_2_O_2_-generating system in neurons during the autophosphorylation process.

**Conclusions:**

In this study, we demonstrated for the first time that the receptor autophosphorylation occurs only if mitochondrial H_2_O_2_ signal exceeds a certain threshold. This finding provides novel insights into the mechanisms underlying neuronal response to insulin. The neuronal insulin receptor is activated if two conditions are met: 1) insulin binds to the receptor, and 2) the H_2_O_2_ signal surpasses a certain threshold, thus, enabling receptor autophosphorylation in all-or-nothing manner. Although the physiological rationale for this control remains to be determined, we propose that malfunction of mitochondrial H_2_O_2_ signaling may lead to the development of cerebral insulin resistance.

## Background

Insulin receptor is a member of the receptor tyrosine kinase family. Upon insulin binding to the extracellular α-subunits, the receptor undergoes rapid autophosphorylation at three specific tyrosine residues within the activation loop of the cytoplasmic β-subunits [1,2], resulting in more than a 200-fold increase in receptor tyrosine kinase activity [3]. Therefore, the autophosphorylated receptor is regarded as fully activated [4]. Research conducted over 30 years ago revealed that cells generate hydrogen peroxide (H_2_O_2_) in response to insulin stimulation [5,6]. Evidence from several studies supports the hypothesis that the main role of insulin-induced H_2_O_2_ is inhibition of protein tyrosine phosphatases (PTPs), which otherwise dephosphorylate the autophosphorylated insulin receptor [7-9]. According to this theory, H_2_O_2_ prolongs the duration of time for which the insulin receptor remains active, rather than directly influence receptor activation. Additionally, exogenous H_2_O_2_ has been shown to facilitate receptor autophosphorylation in immunoprecipitates of the insulin receptor in the presence of phosphate donors [10,11]. The obvious independence of this effect on intracellular PTPs suggests that H_2_O_2_ also participates in insulin receptor activation. Insulin receptors are widely distributed in the brain, where they play roles in synaptic function, memory formation, and neuroprotection [12-14]. The neuron-specific isoform A is the predominant insulin receptor type in the brain. Isoform A is generated from alternative splicing and differs from its peripheral counterpart (isoform B) in some notable respects, such as higher affinity for insulin and absence of negative cooperativity in insulin binding [15,16]. Earlier studies by our group demonstrated that neurons generate H_2_O_2_ in response to insulin stimulation [17]. This H_2_O_2_ is derived from the mitochondrial respiratory chain and plays a role in insulin receptor autophosphorylation. However, the kinetic characteristics of the H_2_O_2_ signal and its functional relationships with the insulin receptor during autophosphorylation in neurons remain to be clarified. In the current investigation, these issues have been explored as an extension of our previous study.

## Results and discussion

### Insulin dose–response for receptor autophosphorylation is well described by hyperbolic function

**Figure 1:**
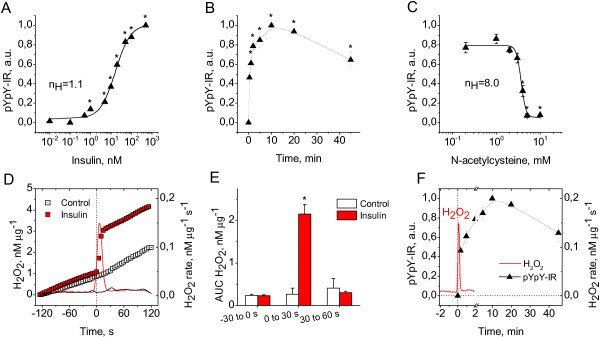
**Relationship between H**_**2**_**O**_**2 **_**signal and receptor autophosphorylation in neurons stimulated with insulin. (A)** Insulin dose–response for receptor autophosphorylation in CGN exposed to insulin for 10 min (black triangles, mean±SEM of 5 to 9 cultures, *P<0.05 vs. control). **(B)** Time course of receptor autophosphorylation in CGN exposed to 100 nM insulin (black triangles, mean±SEM of 3 to 4 cultures, *P<0.05 vs. baseline). **(C)** N-acetylcysteine dose–response for receptor autophosphorylation in CGN exposed to 100 nM insulin for 10 min (black triangles, mean±SEM of 3 to 7 cultures, *P<0.05 vs. 100 nM insulin). **(D)** Left Y axis: time courses of H_2_O_2_ efflux from CGN exposed to vehicle (white squares, mean of 3 culture dishes) or 100 nM insulin (red squares, mean of 10 culture dishes). Right Y axis: first time derivative (rate) of H_2_O_2_ efflux from CGN exposed to vehicle (black line) or 100 nM insulin (red line). **(E)** Areas under curves (AUC) for 30-s periods of H_2_O_2_ efflux from CGN exposed to vehicle (white columns, mean±SEM, n=3) or 100 nM insulin (red columns, mean±SEM, n=10, *P<0.05 vs. control). **(F)** Time courses for insulin-stimulated H_2_O_2_ efflux and receptor autophosphorylation. Left Y axis: time course of receptor autophosphorylation in CGN exposed to 100 nM insulin (black triangles, mean±SEM of 3 to 4 cultures). Right axis: first time derivative (rate) of H_2_O_2_ efflux from CGN exposed to 100 nM insulin (red line, mean of 10 culture dishes).

We characterized insulin-stimulated receptor autophosphorylation in a primary culture of rat cerebellar granule neurons (CGN). The insulin dose–response curve for the autophosphorylation process is depicted in Figure 1A. Fitting the curve to the Hill equation generated ED_50_ of 16.3±2.2 nM and Hill coefficient, n_H_, of 1.1±0.1 (R^2^=0.99), indicating that this process in neurons is described by a classic hyperbolic function. These results suggest that the insulin dose–response for receptor autophosphorylation in neurons is gradual and not switch-like.

### The H_2_O_2_ signal precedes receptor autophosphorylation during insulin stimulation

To determine the temporal relationship between receptor autophosphorylation and H_2_O_2_ generation during insulin stimulation, we compared the kinetics of insulin-induced autophosphorylation and H_2_O_2_ production in CGN. The time-course of receptor autophosphorylation in response to insulin stimulation is depicted in Figure 1B. Autophosphorylation peaked at 10 min and dissipated by 30% at 45 min of stimulation. Figure 1D presents the kinetics of the insulin-induced H_2_O_2_ signal. H_2_O_2_ efflux from neurons into the incubation medium was used as a measure of the signal, given that H_2_O_2_ penetrates readily across cellular membranes with an estimated time of gradient formation within 1 s [18]. Our data showed that insulin stimulation evokes a transient single H_2_O_2_ spike with a peak at 5–10 s and duration of less than 30 s. We observed a significant difference between the areas under curves (AUCs) calculated for the H_2_O_2_ signal in insulin- and vehicle-exposed neurons during the 30 s of insulin stimulation (*P*<0.001), while no difference was observed at baseline and periods following this time (Figure 1E). A comparison of the kinetic curves for the insulin-induced H_2_O_2_ signal and receptor autophosphorylation revealed that the H_2_O_2_ signal precedes autophosphorylation (Figure 1F). Notably, at 20 s of stimulation, when the H_2_O_2_ signal was 95% complete, receptor autophosphorylation was still in progress.

### The H_2_O_2_ signal generates strong ultrasensitivity in insulin-induced receptor autophosphorylation

To address the specific function of the H_2_O_2_ signal in insulin-stimulated receptor autophosphorylation, we investigated the effects of increasing concentrations of N-acetylcysteine (NAC), a scavenger of hydrogen peroxide, on receptor autophosphorylation in neurons stimulated with insulin. NAC dose-dependently and completely (at concentrations ≥4 mM) inhibited receptor autophosphorylation in CGN cultures exposed to 100 nM insulin (Figure 1C). Approximation of the experimental dose–response data with the Hill function generated IC_50_ of 3.7±0.2 mM and a Hill coefficient, n_H_, of 8.0±2.3 (R^2^=0.97), signifying that the observed dose response is highly sigmoidal and considerably steeper than a hyperbolic curve (n_H_=1). Therefore, even a small increase in the H_2_O_2_ scavenger dose above the threshold results in complete abrogation of receptor autophosphorylation. Given that a steep dose–response with a Hill coefficient n_H_>1 is defined as ultrasensitive [19,20], the results suggest that the insulin-induced H_2_O_2_ signal generates strong ultrasensitivity in receptor autophosphorylation. Autophosphorylation only occurs when the H_2_O_2_ signal has surpassed a certain threshold. Conversely, if the H_2_O_2_ signal does not reach this threshold, no autophosphorylation occurs, even in response to the highest insulin dose (100 nM). Therefore, H_2_O_2_ appears to function as an enabling signal that permits insulin receptor autophosphorylation in an all-or-nothing manner. This mode of switching between two modes of action is often referred to as a decision-making process. In this context, H_2_O_2_ signal above the threshold serves as a decision-making step of the neuron to permit activation of the insulin receptor.

### Succinate dehydrogenase of mitochondrial complex II regulates the insulin-induced H_2_O_2_ signal

**Figure 2:**
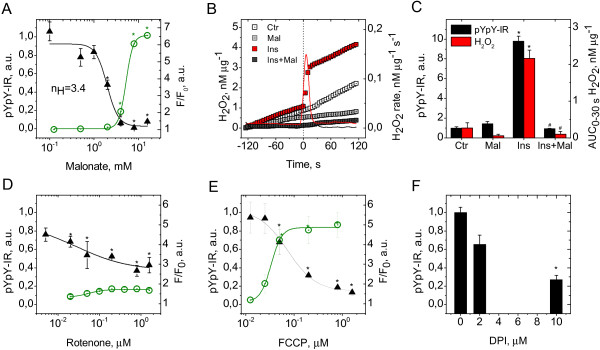
**Effects of inhibitors on H**_**2**_**O**_**2 **_**signal and receptor autophosphorylation in neurons stimulated with 100 nM insulin. (A)** Left Y axis: malonate dose–response for receptor autophosphorylation in CGN exposed to insulin (triangles, mean±SEM of 3-11 cultures, *P<0.05 vs. insulin). Right Y axis: malonate effect on Rhodamine 123 fluorescence in CGN (circles, mean±SEM of 3-7cultures, *P<0.05 vs. control). **(B)** Left Y axis: kinetics of H_2_O_2_ efflux from CGN exposed to vehicle (mean of 3 cultures), 6 mM malonate (mean of 3 cultures), insulin (mean of 10 cultures), or insulin plus 6 mM malonate (mean of 5 cultures). Right Y axis: rates of H_2_O_2_ efflux from CGN exposed to insulin (red line) or insulin plus 6 mM malonate (grey line). **(C)** Left Y axis: receptor autophosphorylation in CGN exposed to vehicle, 6 mM malonate, insulin, or insulin plus 6 mM malonate (mean±SEM of 5-11 cultures. *P<0.05 vs. control. ^#^*P*<0.05 vs. insulin). Right Y axis: areas under curves of H_2_O_2_ efflux from CGN exposed to vehicle, 6 mM malonate, insulin, or insulin plus 6 mM malonate for 30 s (mean±SEM of 3-10 cultures. *P<0.05 vs. control. ^#^*P*<0.05 vs. insulin). **(D)** Left Y axis: rotenone dose–response for receptor autophosphorylation in CGN exposed to insulin (triangles, mean±SEM of 3-4 cultures, *P<0.05 vs. insulin). Right Y axis: rotenone effect on Rhodamine 123 fluorescence in CGN (circles, mean±SEM of 3-4 cultures, *P<0.05 vs. control). **(E)** Left Y axis: FCCP dose–response for receptor autophosphorylation in CGN exposed to insulin (triangles, mean±SEM of 3-5 cultures, *P<0.05 vs. insulin). Right Y axis: FCCP dose-response for Rhodamine 123 fluorescence in CGN (circles, mean±SEM of 4-8 cultures, *P<0.05 vs. control). **(F)** DPI effect on receptor autophosphorylation in CGN exposed to insulin (mean±SEM of 3 cultures, *P<0.05 vs. insulin).

To extend our previous finding that the mitochondrial electron transport chain (ETC.) provides the source of insulin-induced H_2_O_2_ in neurons [17], we investigated the roles of ETC. complexes I and II in this process. Malonate, a competitive inhibitor of succinate dehydrogenase of complex II, dose-dependently and completely (at concentrations ≥ 4 mM) inhibited receptor autophosphorylation stimulated by 100 nM insulin in neurons, as shown in Figure 2A. Approximation of the malonate dose–response curve for autophosphorylation with the Hill function generated an IC_50_ of 2.0±0.3 mM and Hill coefficient, n_H_, of 3.4 (R^2^=0.92), indicating that the dose response is a highly sigmoidal, and therefore, even a small change in succinate dehydrogenase activity around a certain threshold can have a dramatic effect on insulin receptor autophosphorylation. At concentrations of malonate that completely inhibited receptor autophosphorylation, the insulin-induced H_2_O_2_ signal was abolished (Figure 2B,C). Since the inhibitory effects of malonate (on both autophosphorylation and H_2_O_2_ generation) were so complete, we conclude that the reducing equivalents for generation of H_2_O_2_ do not originate from sources other than the succinate dehydrogenase reaction. The results collectively suggest that activation of succinate dehydrogenase is a possible mechanism underlying insulin-mediated switching of the mitochondrial H_2_O_2_ signal critical for receptor autophosphorylation.

As shown in Figure 2B, the rate of H_2_O_2_ generation exhibits biphasic behavior, showing a fast initial increase followed by a rapid decrease to the baseline steady-state level while insulin stimulation is sustained. In keeping with common theory, such a response can be generated by an incoherent feed-forward loop (IFFL) in which insulin modulates the H_2_O_2_ rate via at least two intermediate pathways with opposite functions of “fast activation” and “delayed inhibition”. We speculate that fast activation and delayed inhibition of succinate dehydrogenase generates the biphasic response. In this scheme, delayed inhibition of succinate dehydrogenase is attributed to the downstream metabolites of succinate oxidation, oxaloacetate [21] and H_2_O_2_[22-24]. These metabolites act in orchestrated manner to yield optimal inhibition, since oxaloacetate binds the oxidized form of succinate dehydrogenase more effectively than the reduced form [25] and oxidative conditions induce more significant inhibition of succinate dehydrogenase by oxaloacetate [26-29]. Given that the transition times between the active and inactive states of succinate dehydrogenase [26,30] correspond well to the timing of the insulin-induced biphasic response, we propose that succinate dehydrogenase has sufficient regulatory capacity to generate the transient H_2_O_2_ signal in response to insulin stimulation.

To clarify the factors influencing generation of the insulin-induced H_2_O_2_ signal, we briefly reviewed current knowledge on succinate-supported H_2_O_2_ generation in isolated mitochondria. Succinate promotes the highest rates of H_2_O_2_ production among all respiratory substrates [31-35]. Most mitochondrial H_2_O_2_ is produced from superoxide generated by reduction of molecular oxygen in the electron transport chain (ETC.) [36,37]. Although several ETC. sites may generate superoxide in mitochondria respiring on succinate, the majority is produced at complex II [38] and at complex I during reverse electron transport (RET) from complex II [32-36], where complex I-associated superoxide production is sensitive to modulation of the mitochondrial membrane potential, ΔΨ_m_[32,39]. Notably, RET-associated superoxide generation is observed at the highest non-physiological succinate concentrations at which superoxide production at complex II is suppressed, whereas generation at complex II occurs only within the range of lower physiologically relevant succinate levels [38]. At succinate concentrations favoring RET-associated H_2_O_2_ production, generation of H_2_O_2_ depends on the metabolic state of mitochondria and changes in parallel with succinate dehydrogenase activity. The highest H_2_O_2_ rates [37] and succinate dehydrogenase activity [27,28] are observed at metabolic state 4. On transition to state 3, H_2_O_2_ production rates drop rapidly [34,39] and succinate dehydrogenase activity decreases [27,28]. Protonophores completely inhibit H_2_O_2_ production [33,39] and deactivate succinate dehydrogenase to the lowest observable activity [27]. In summary, the documented literature suggests that ΔΨ_m_ and activity of mitochondrial complexes I/II are the candidate factors that influence generation of the insulin-induced H_2_O_2_ signal and thus play a role in the control of insulin-stimulated receptor autophosphorylation in neurons.

### Mitochondrial complex I is involved in control of insulin-induced receptor autophosphorylation, but to a lower extent than complex II

To investigate whether ETC. complex I functions in the control of insulin-stimulated receptor autophosphorylation, we examined the effects of rotenone on receptor autophosphorylation in neurons stimulated with 100 nM insulin. Rotenone is a selective inhibitor of the ubiquinone reduction site at mitochondrial complex I. Rotenone inhibited insulin-stimulated receptor autophosphorylation, as shown in Figure 2D. The effect of rotenone was significant, but incomplete, and even at the highest concentration, autophosphorylation was reduced by less than 50%. Fitting the dose–response curve generated a Hill coefficient, n_H_, of 0.7 (R^2^=0.83), indicating that the observed dose response is gradual and not switch-like. Our results suggest that ETC. complex I is involved in control of insulin receptor autophosphorylation, presumably as part of the mitochondrial machinery of RET-associated H_2_O_2_ generation. However, its role does not appear to be as important as that of ETC. complex II.

### Insulin-induced receptor autophosphorylation is sensitive to mitochondrial depolarization

To address whether receptor autophosphorylation is sensitive to ΔΨ_m_, we compared the effects of insulin and other additives on autophosphorylation and ΔΨ_m_ in neurons. Rhodamine 123 was used as a measure of mitochondrial ΔΨ_m_[40]. Insulin did not have a significant effect on Rhodamine 123 fluorescence in neurons. Malonate induced a dose-dependent increase in Rhodamine 123 fluorescence, signifying that inhibition of succinate dehydrogenase results in mitochondrial depolarization (Figure 2A). Fitting the fluorescence curve to the Hill equation led to ED_50_ of 5.2±0.1 mM and Hill coefficient, n_H_, of 5.4 (R^2^=0.99). Comparison of the malonate dose–response curves for autophosphorylation and fluorescence revealed that at a malonate concentration of 2 mM (inducing 50% inhibition of autophosphorylation), mitochondria were depolarized by less than 1%. This finding suggests that ΔΨ_m_ in neurons is ultrasensitive to succinate dehydrogenase activity and mitochondrial depolarization is not a causative factor for the inhibitory effect of malonate on receptor autophosphorylation. Figure 2D shows that the inhibitory effect of rotenone on autophosphorylation is not accompanied by mitochondrial depolarization. The protonophore, FCCP, known to dissipate the transmembrane proton gradient, evoked an increase in Rhodamine 123 fluorescence and inhibited insulin-stimulated receptor autophosphorylation in a dose-dependent manner (Figure 2E). Fitting the FCCP dose–response curves for fluorescence and autophosphorylation gave ED_50_ of 0.030±0.003 (R^2^=0.99) and IC_50_ of 0.07±0.02 (R^2^=0.98), respectively. At a FCCP concentration of 0.07 μM (inducing 50% inhibition of autophosphorylation), >95% mitochondrial depolarization was observed. These data suggest that FCCP-induced mitochondrial depolarization leads to inhibition of insulin-stimulated receptor autophosphorylation. Given that protonophores deactivate succinate dehydrogenase to the lowest observable activity [27], we propose that the inhibitory effect of depolarization on autophosphorylation is mediated via deactivation of succinate dehydrogenase. Taken together, these results suggest that insulin receptor autophosphorylation is sensitive to factors inducing mitochondrial depolarization, while modulation of ΔΨm is not implicated in the mechanism of insulin-triggered receptor autophosphorylation.

### Nox is not involved in control of insulin-induced receptor autophosphorylation in neurons

A number of previous studies using non-neuronal cells have assigned the insulin-induced H_2_O_2_ signal to activation of NADPH oxidase (Nox) that is sensitive to inhibition by diphenyleneiodonium (DPI) [41-43]. Accordingly, we investigated the effect of DPI on receptor autophosphorylation in neurons exposed to 100 nM insulin. DPI treatment at a concentration of 10 μM significantly inhibited insulin-stimulated receptor autophosphorylation. Moreover, the inhibitory action of DPI was dose-dependent (Figure 2F). Since selective mitochondrial inhibitors that do not inhibit Nox completely abrogate both insulin-induced H_2_O_2_ generation and receptor autophosphorylation, the results of the DPI experiment cannot be interpreted to conclude that Nox is a source of the insulin-induced H_2_O_2_ signal in neurons. Although the nonspecific flavoprotein inhibitor, DPI, is often claimed to be a specific inhibitor of Nox, compelling evidence shows that DPI inhibits many targets [44], including ROS generation at the mitochondrial complex I [45,46] and succinate-supported H_2_O_2_ generation in rat brain mitochondria [33]. In this context, data from the DPI experiment do not contradict our main conclusion that the mitochondrial ETC. is the only source of insulin-induced H_2_O_2_ in neurons. Another argument against a role of Nox in neuronal insulin receptor autophosphorylation is that the kinetics of the insulin-induced H_2_O_2_ signal is unusually fast, compared to that previously observed for Nox in non-neuronal cells [8,43]. The neuronal H_2_O_2_ signal is a single spike with a sharp peak at 5–10 s and duration of less than 30 s. In contrast, the insulin-induced oxidant signal in adipocytes reaches a peak at 5 min and begins to dissipate by 10 min [8]. In HepG2 cells, ROS generation is optimal at about 10 min and dissipates after 30 min of insulin stimulation [43]. This significant difference in the timing of H_2_O_2_ signals indicates that the source of insulin-induced H_2_O_2_ in neurons differs from that in adipocytes and HepG2 cells, which is Nox [8,43]. Our results collectively suggest that Nox is not involved in H_2_O_2_ signaling that controls insulin-stimulated receptor autophosphorylation in neurons.

Data from the present study raise the issue of whether the mitochondrial origin of the insulin-induced H_2_O_2_ signal is a neuron-specific phenomenon. Although abundant literature is available on insulin-induced H_2_O_2_ generation in cells, limited studies have focused on the origin of insulin-induced H_2_O_2_ implicated in insulin receptor autophosphorylation. In experiments with 3T3-L1 adipocytes [41,42] and HepG2 cells [43], the source of H_2_O_2_ was assigned to activated Nox on the basis of data obtained with DPI only. Strong evidence from experiments with dominant-negative Nox4 constructs and Nox4 siRNA further suggested that Nox4 is the source of the insulin-induced H_2_O_2_ signal in adipocytes [47]. The results from the present study, together with previous data [17], indicate that mitochondrial ETC. is the only source of insulin-induced H_2_O_2_ in neurons. In summary, owing to limited data, it is not currently possible to address whether mitochondria represent the only neuronal source of insulin-induced H_2_O_2_ implicated in receptor activation.

### The insulin-induced H_2_O_2_ signal and receptor autophosphorylation in neurons are pertussis toxin-sensitive

**Figure 3:**
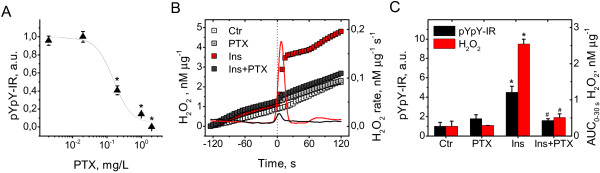
**Effects of pertussis toxin on H**_**2**_**O**_**2 **_**generation and receptor autophosphorylation neurons stimulated with insulin. (A)** PTX dose–response curve for receptor autophosphorylation in CGN exposed to 100 nM insulin for 10 min (black triangles, mean±SEM of 3 to 8 cultures, *P<0.05 vs. 100 nM insulin). **(B)** Left Y axis: time courses of H_2_O_2_ efflux from CGN exposed to control buffer (white squares, mean of 3 culture dishes), 2 mg/L PTX (light grey squares, mean of 3 culture dishes), 100 nM insulin (red squares, mean of 3 culture dishes), or 100 nM insulin plus 2 mg/L PTX (grey squares, mean of 3 culture dishes). Right Y axis: first time derivatives (rates) of H_2_O_2_ efflux from CGN exposed to 100 nM insulin (red line) or 100 nM insulin plus 2 mg/L PTX (black line). **(C)** Left Y axis: receptor autophosphorylation in CGN exposed to control buffer, 2 mg/L PTX, 100 nM insulin, or 100 nM insulin plus 2 mg/L PTX. Black columns represent the mean±SEM of values obtained from 3 to 8 cultures. *P<0.05 vs. control. ^#^*P*<0.05 vs. insulin. Right Y axis: Areas under curves (AUC) of H_2_O_2_ efflux for 30 s from CGN exposed to control buffer, 2 mg/L PTX, 100 nM insulin, or 100 nM insulin plus 2 mg/L PTX. Red columns represent the mean±SEM of values obtained from 3 culture dishes. *P<0.05 vs. control. ^#^*P*<0.05 vs. insulin.

A pertussis toxin-sensitive G protein links the insulin receptor to the insulin-induced H_2_O_2_ signal in non-neuronal cells [48]. To determine whether G protein signaling is involved in generation of insulin-induced H_2_O_2_ in neurons, we examined the effects of pertussis toxin (PTX), an inhibitor of Gi/0 protein-receptor coupling, on H_2_O_2_ generation and receptor autophosphorylation in CGN exposed to 100 nM insulin. PTX treatment completely inhibited insulin-stimulated receptor autophosphorylation at the highest dose of 2 mg/L, and this suppression was dose-dependent (Figure 3A). Approximation of the curve to the Hill equation gave IC_50_ of 0.16±0.06 mg/L and Hill coefficient, n_H_, of 1.7±2.0 (R^2^=0.96), implying that insulin-induced receptor autophosphorylation is ultrasensitive to PTX. PTX (2 mg/L) completely eliminated the insulin-induced H_2_O_2_ signal (Figure 3B). Thus, at the dose at which PTX completely abrogated the H_2_O_2_ signal (2 mg/L), receptor autophosphorylation was abolished, as shown in Figure 3C. Based on these results, we suggest that a PTX-sensitive G protein links the insulin receptor to the H_2_O_2_-generating system in neurons during generation of the insulin-induced H_2_O_2_ signal critical for insulin receptor autophosphorylation.

Although our data show for the first time that a PTX-sensitive G protein is possibly involved in activation of the insulin receptor in CNS, the involvement of a PTX-sensitive G protein in insulin receptor activation in peripheral tissues is widely documented. In mice, Gαi2 deficiency results in impaired tyrosine phosphorylation of the insulin receptor substrate, IRS1, and frank insulin resistance in peripheral tissues [49], whereas targeted expression of a constitutively active form of Gαi2 enhances insulin action through amplifying tyrosine phosphorylation of the insulin receptor and IRS1 [50-52]. In human adipocytes, insulin recruits Gαi2 to activate Nox-dependent H_2_O_2_ generation [48] and regulate insulin receptor autophosphorylation [53]. Although no data supporting the involvement of Gαi2 in brain insulin signaling are available, Gαi2, and especially its short-lived splice variant, sGi2, are widely distributed throughout rat and monkey brain, where sGi2 is detected in both axons and dendrites at presynaptic and postsynaptic sites [54]. Gαi2 operates largely in plasma membranes, while sGi2 is localized in a variety of subcellular locations, including mitochondria [54-56]. Our present results are generally in keeping with current knowledge on the amplification role of pertussis toxin-sensitive Gi protein in insulin receptor activation. However, the specific G protein isoform implicated in neuronal insulin receptor activation remains to be determined.

### Functional association of the insulin receptor and mitochondria during receptor activation in neurons

**Figure 4:**
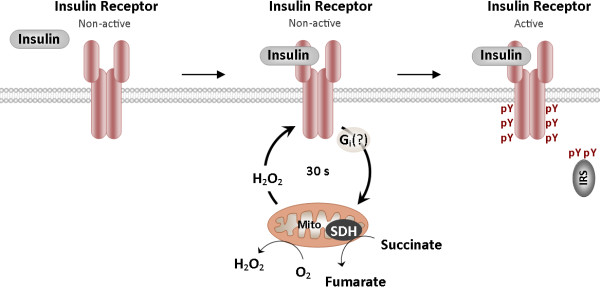
**Scheme of functional relationship between insulin receptor and mitochondria during receptor activation in neurons.** Insulin stimulation evokes a transient single H_2_O_2_ spike with a peak at 5–10 s and duration of less than 30 s. Mitochondrial complex II and, to a lesser extent, I are involved in H_2_O_2_ generation. Autophosphorylation only occurs when the H_2_O_2_ signal has surpassed a certain threshold. Conversely, if the H_2_O_2_ signal does not reach this threshold, no autophosphorylation occurs, even in response to the highest insulin dose. Upon the autophosphorylation, receptor tyrosine kinase becomes fully activated and initiates signaling to the inside of the neuron. Therefore, the receptor is activated if two conditions are met: 1) insulin binds to the receptor, and 2) the H_2_O_2_ signal exceeds a certain threshold, enabling receptor autophosphorylation.

It is possible to draw some tentative conclusions regarding the functional relationship between the insulin receptor and mitochondria during receptor activation in neurons (Figure 4). Insulin stimulation induces receptor autophosphorylation, which reaches a peak at 10 min. Upon autophosphorylation, the receptor becomes fully activated and initiates signaling to the inside of the neuron. At times preceding autophosphorylation, insulin induces a transient H_2_O_2_ signal, which plays a permissive role in activation of the insulin receptor. Autophosphorylation only occurs once the H_2_O_2_ signal has surpassed a certain threshold. Under conditions where the H_2_O_2_ signal does not reach this threshold, no autophosphorylation occurs, even in response to the highest insulin dose. In this context, H_2_O_2_ signal above the threshold serves as the neuron’s decision to activate the insulin receptor. The insulin-induced H_2_O_2_ signal is derived from mitochondria. Succinate dehydrogenase in complex II plays a key role in control of H_2_O_2_ generation. An unknown pertussis toxin-sensitive G protein links the insulin receptor to the mitochondrial H_2_O_2_-generating system during H_2_O_2_ signal activation.

The current study provides novel insights into the mechanisms underlying neuronal response to insulin. The insulin receptor is activated if two conditions are met: 1) insulin binds to the receptor, and 2) the H_2_O_2_ signal exceeds a certain threshold, enabling receptor autophosphorylation. Generally, this mode of control means that activation of the neuronal insulin receptor is conditional on mitochondrial functioning. Moreover, receptor activation may be conditional on neural activity. Neural activity evokes insulin release from synaptosomes within nerve endings into the synaptic cleft [57,58] and induces mitochondrial migration to dendritic spines [59], which are commonly poor in mitochondria. Therefore, periods of synaptic activity favor insulin receptor activation in the postsynaptic density of dendritic spines where the two above conditions are met.

### Possible links between impairment of H_2_O_2_ signaling and insulin resistance

Given the importance of the insulin-induced H_2_O_2_ signal in autophosphorylation of the neuronal insulin receptor, factors that disrupt H_2_O_2_ signaling may generate unresponsiveness or resistance to insulin. In line with the above findings, mitochondrial dysfunction that affects generation of the H_2_O_2_ signal may induce central insulin resistance. Moreover, insulin resistance may be induced by pathologically elevated activity of antioxidant enzymes that metabolize H_2_O_2_, e.g., arising as a compensatory response to oxidative stress. Accumulating evidence from animal and human studies supports this hypothesis. Glutathione peroxidase (Gpx1) and peroxiredoxins (Prx) are primary H_2_O_2_ scavengers capable of metabolizing the insulin-induced H_2_O_2_ signal, according to kinetic estimations based on published second-order rate constants of ~10^7^ M^-1^ s^-1^ and abundance data [60-62]. Mice overexpressing Gpx1 are insulin-resistant, obese, and along with hyperinsulinemia, display a 70% reduction in insulin-stimulated phosphorylation of insulin receptors, compared to wild-type control mice [63]. In contrast, mice lacking Gpx1 are protected from high fat diet-induced insulin resistance, while administration of the H_2_O_2_ scavenger, NAC, renders them more insulin-resistant [64]. Brain insulin resistance is an early and common feature of Alzheimer’s disease (AD). Compared to age-matched normal brains, AD brains are characterized by diminished tyrosine phosphorylation of the insulin receptor and its substrate, IRS1 [65], and significant overexpression of glutathione peroxidase [66,67] and peroxiredoxins (Prx1 and Prx2) [68-71]. Elevated antioxidant activity in early AD is considered the result of compensatory response to oxidative stress [72,73]. From this viewpoint, oxidative stress seems to be another factor that interferes with the insulin-induced H_2_O_2_ signal and consequently induces central insulin resistance.

## Conclusions

In summary, we have demonstrated for the first time that the receptor autophosphorylation occurs only if mitochondrial H_2_O_2_ signal exceeds a certain threshold. Our findings provide a novel insight into the mechanisms underlying neuronal response to insulin. The neuronal insulin receptor is activated if two conditions are met: 1) insulin binds to the receptor, and 2) the H_2_O_2_ signal surpasses a certain threshold, thus, enabling receptor autophosphorylation in all-or-nothing manner. Although the physiological rationale for this control remains to be determined, we propose that malfunction of insulin-induced H_2_O_2_ signaling may lead to cerebral insulin resistance, in view of the critical significance of the H_2_O_2_ signal for insulin receptor activation in neurons.

## Methods

### Materials

PhosphoDetect™ Insulin Receptor (pTyr1162/1163) ELISA kit and Insulin Receptor (β-Subunit) ELISA Kit were from Calbiochem. Other materials were purchased from Sigma, ICN, Gibco, Biosource, Invitrogen and Acros.

### Neuronal culture

Cerebellar granule neurons were prepared from 7- to 8-day-old Wistar rats, as described earlier [74]. Cerebellum was dissected and placed in ice-cold Ca^2+^/Mg^2+^-free Hanks’ buffered salt solution (HBSS) without Phenol Red (Gibco). After mincing with fine scissors, tissue was placed in Ca^2+^/Mg^2+^-free HBSS with Phenol Red and 0.1% trypsin for 15 min at 37°C. Trypsin was inactivated by washing with normal HBSS. Cells were dissociated via trituration and pelleted in HBSS. Next, cells were resuspended in Neurobasal Medium (Gibco) supplemented with serum-free B-27 Supplement (100X, Gibco), 20 mmol/L KCl, GlutaMax (100X, Gibco) and antibiotic-antimycotic (100X, Gibco), and plated at a density of 5×10^6^ cells/ml onto 35X10 mm sterile cell culture dishes or 6×24 plates (Corning) previously coated with polyethyleneimine (10 mkg/ml). Cultures were maintained at 37°C in a humidified atmosphere of 5% CO_2_ and 95% air, and fed with supplemented Neurobasal Medium. Cultures were treated on day 3 with 5 μM cytosine arabinoside (Sigma) for 24 h to prevent glial proliferation, and the medium subsequently changed. Neurons on days 7 to 9 were used for experiments.

### Measurement of hydrogen peroxide

H_2_O_2_ efflux from CGN cultures was measured using fluorimetry employing the cell-impermeable Amplex Red dye in the presence of horseradish peroxidase (Amplex^®^ Red Hydrogen Peroxide/Peroxidase Assay Kit, Cat. No. A22188, Invitrogen). CGN cultures were pre-incubated for 120 min in Hepes-buffered salt solution composed of 156 mM NaCl, 3 mM KCl, 2 mM MgSO_4_, 1.25 mM KH_2_PO_4_, 2 mM CaCl_2_, 0.5 mM glucose, 20 mM HEPES, and the pH adjusted to 7.4 with NaOH. Cultures were subsequently exposed to Hepes-buffered salt solution supplemented with 50 μM Amplex Red and 0.1 U/ml horseradish peroxidase (incubation medium), and H_2_O_2_ efflux recorded every 5 s for 2 min. The incubation medium was rapidly exchanged with fresh medium containing no insulin (control) or 100 nM insulin, and H_2_O_2_ efflux recorded every 5 s for 2 min. Where indicated, PTX (2 μg/ml) was added 120 min before and during insulin stimulation. Malonate (6 mM) was added 5 min before and during insulin stimulation. Fluorescence was measured with an epifluorescent inverted microscope Axiovert 200 (Carl Zeiss, Germany) equipped with a 20X fluorite objective, using excitation at 550±10 nm and fluorescence detection at 610±30 nm. All imaging data were collected and analyzed using Metafluor 6.1 software (Universal Imaging Corp., USA). Standard curves of H_2_O_2_ concentrations were linear up to 1500 nM and used to convert fluorescence values to H_2_O_2_ values. The calculated detection limit of the assay was 7 nM. Data were normalized to total amounts of cell protein, and expressed as H_2_O_2_ nM μg^-1^.

### Insulin receptor autophosphorylation assay

Amounts of the autophosphorylated β-subunit of the insulin receptor were measured with the PhosphoDetect™ insulin receptor (pTyr1162/1163) ELISA kit (Calbiochem) suitable for studies with the rat insulin receptor. CGN cultures were incubated in Hepes-buffered salt solution (145 mM NaCl, 5.6 mM KCl, 1.8 mM СаCl_2_, 1 mM MgCl_2_, 20 mM HEPES, and 0.5 mM glucose) at pH 7.4 for 30 min, followed by exposure to insulin (10 min, 37°C) or no insulin (control) in the presence or absence of other additives (preincubation for 5–30 min before insulin). The experiment was terminated by removing the medium, washing with ice-cold PBS, and adding 200 μL/dish cell lysis buffer (Biosource) supplemented with 1 mM PMSF, 50 mM protease inhibitor set III (Sigma), and 2 mM sodium orthovanadate as the tyrosine phosphatase inhibitor on ice for 10 min. Lysates were centrifuged at 12,000 rpm at 4°C for 12 min. In each CGN lysate, amounts of autophosphorylated receptors were measured as described by the manufacturer. For each experiment, values were normalized to the total amounts of insulin receptor β-subunit (IR) measured using the insulin receptor (β-subunit) ELISA kit (Calbiochem) and normalized to scale between 0 and 1 using Equation 1:

(1) $$\text{Normalized}\left(Y_{i}\right)=\frac{Y_{i}-Y_{\text{CTR}}}{Y_{\text{INS}}-Y_{\text{CTR}}}$$

where *Y*_*i*_ is an original receptor autophosphorylation value, *Normalized (Y*_*i*_*)* the normalized autophosphorylation value, *Y*_*CTR*_ the mean autophosphorylation value in neurons exposed to vehicle, and *Y*_*INS*_ the mean autophosphorylation value in neurons exposed to 100 nM insulin.

### Monitoring of Rhodamine 123 fluorescence

Mitochondrial depolarization changes in individual neurons within CGN cultures in response to additives were measured via fluorescence of Rhodamine 123, as described earlier [75]. CGN cultures were equilibrated with 10 μg/ml Rhodamine 123 in Hepes-buffered salt solution for 10 min at 20°C. Cells were washed with HBSS before the experiment (excitation, 488 nm; emission, 530 nm). CGN cultures were exposed to insulin, rotenone, malonate or FCCP for 10 min, and fluorescence measured. Data were expressed as the F/F_0_ ratio of the fluorescence signal measured after exposure of neurons to additives to that measured at baseline.

### Curve fitting

Experimental data were fitted by non-linear regression to the Hill equation to provide the parameter values in Equation 2:

(2) $$Y=\text{Start}+\frac{\left(\text{End}-\text{Start}\right)\times X^{n_{H}}}{K^{n_{H}}+X^{n_{H}}}$$

where *Y* is a parameter value, *X* a variable, *K* the concentration of the variable producing half the effect, and *n*_*H*_ the Hill coefficient.

### Statistical analysis

Data were analyzed for statistical significance with one-way analysis of variance (ANOVA). Values are presented as means ± SEM. Differences were considered significant at *P*<0.05.

## Abbreviations

CGN: Cerebellar granule neurons; DPI: Diphenyleneiodonium; FCCP: Carbonyl cyanide-4-(trifluoromethoxy)-phenylhydrazone; HBSS: Hanks’ buffered salt solution; HEPES: 4-(2-hydroxyethyl)-1-piperazineethanesulfonic acid; NAC: N-acetylcysteine; PBS: Phosphate-buffered saline; PMSF: Phenylmethylsulfonyl fluoride; PTX: Pertussis toxin; PTPs: Protein tyrosine phosphatases; SEM: Standard error of mean.

## Competing interests

The authors declare that they have no competing interests.

## Authors’ contributions

NAP carried out the *in vitro* studies with CGN cultures and data analysis. TPS carried out the in vitro studies with CGN cultures and data analysis. YES carried out the in vitro studies with CGN cultures and data analysis. LRG carried out the DPI experiments in CGN cultures and data analysis; VGP participated in the design of the in vitro studies with CGN cultures, and manuscript evaluation/critique. IAP conceived, designed and coordinated the study, and drafted the manuscript. All authors read and approved the final manuscript.
